# Social Capital, Black Social Mobility, and Health Disparities

**DOI:** 10.1146/annurev-publhealth-052020-112623

**Published:** 2022-01-06

**Authors:** Keon L. Gilbert, Yusuf Ransome, Lorraine T. Dean, Jerell DeCaille, Ichiro Kawachi

**Affiliations:** 1 Department of Behavioral Health Science and Health Education, College for Public Health and Social Justice, Saint Louis University, St. Louis, Missouri, USA;; 2 Governance Studies, The Brookings Institution, Washington, DC; 3 Department of Social and Behavioral Sciences, School of Public Health, Yale University, New Haven, Connecticut, USA;; 4 Department of Epidemiology, Bloomberg School of Public Health, Johns Hopkins University, Baltimore, Maryland, USA;; 5 Department of Social and Behavioral Sciences, Harvard T.H. Chan School of Public Health, Harvard University, Cambridge, Massachusetts, USA;

**Keywords:** social capital, social mobility, racial disparities, structural racism

## Abstract

This review aims to delineate the role of structural racism in the formation and accumulation of social capital and to describe how social capital is leveraged and used differently between Black and White people as a response to the conditions created by structural racism. We draw on critical race theory in public health praxis and restorative justice concepts to reimagine a race-conscious social capital agenda. We document how American capitalism has injured Black people and Black communities’ unique construction of forms of social capital to combat systemic oppression. The article proposes an agenda that includes communal restoration that recognizes forms of social capital appreciated and deployed by Black people in the United States that can advance health equity and eliminate health disparities. Developing a race-conscious social capital framing that is inclusive of and guided by Black community members and academics is critical to the implementation of solutions that achieve racial and health equity and socioeconomic mobility.

## INTRODUCTION

This article begins with framing the Black American freedom struggle, which may be broadly defined as a social movement or social activism to achieve racial equity. This movement within Black communities has mobilized different forms of social capital as a strategy to promote social opportunity and redress inequities created by structural racism. Black Americans have leveraged unique forms of social capital to work toward closing the persistent gaps in education, health, and economic mobility; however, these contributions have not been sufficiently recognized or appreciated in the social capital literature (18, 36). Advancing health equity within public health requires a race-conscious social capital framing to eliminate health inequities by radically engaging Black communities in particular.

## STRUCTURAL RACISM AND THE TRUNCATION OF BLACK SOCIAL MOBILITY

This contemporary moment in history (beginning in 2020) is marked by great social, economic, and public health challenges—a global pandemic not seen in a century and racial and civil unrest resulting from state-sanctioned violence. These incredible challenges have sparked unprecedented social activism and declarations that racism constitutes a public health crisis (4, 114).

**Figure 1:**
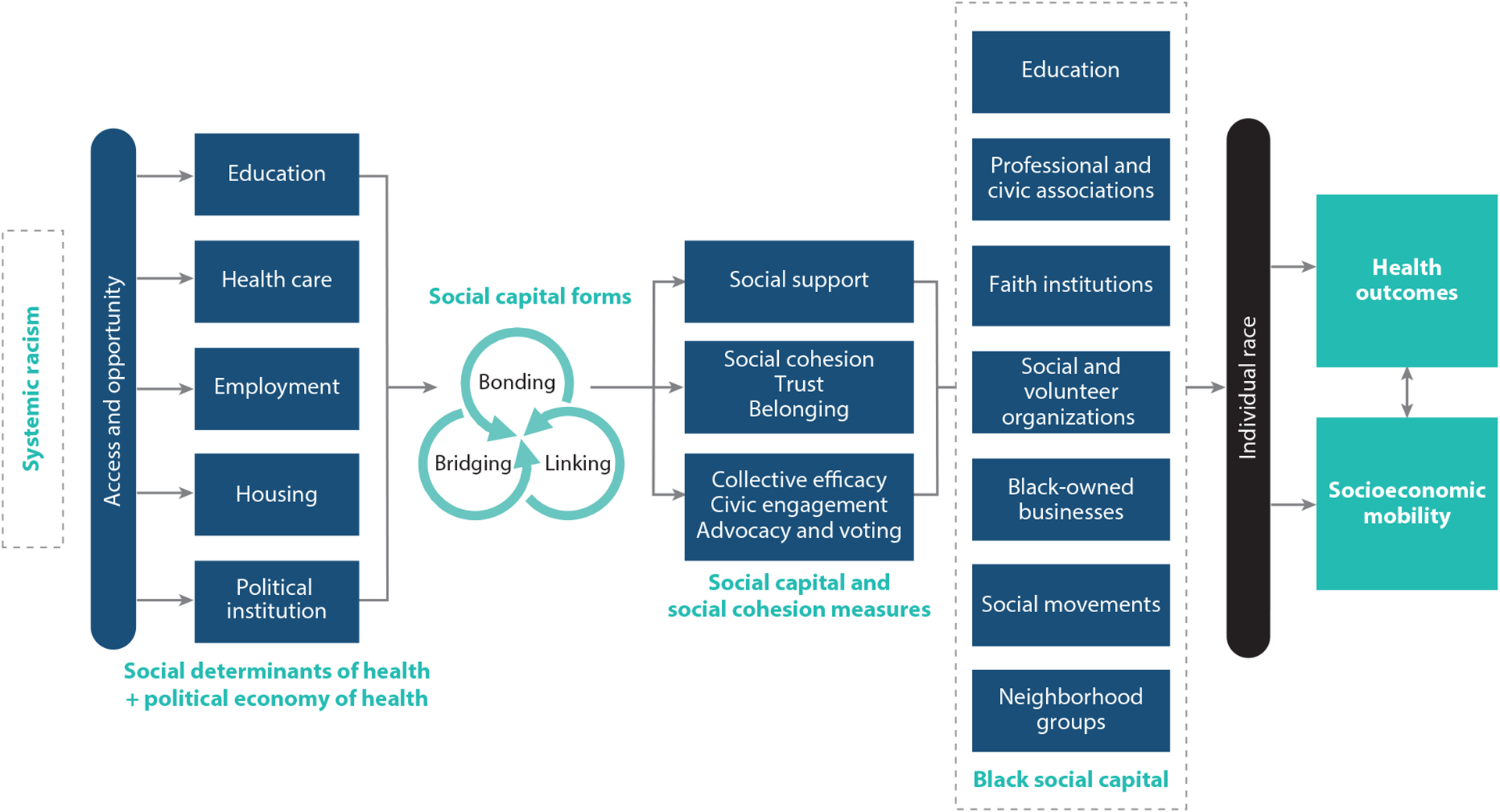
Model for Black social capital and social mobility in Black communities. This model illustrates that systemic racism is the root causal factor working through structures that support access or restrict access to social and political determinants of health. These structures influence the forms and measures of social capital and the recognition of an explicit need to create and acknowledge social capital opportunities created through a Black person’s or Black community’s experience in America. All forms of social capital are subsequently filtered through social constructions of individual race that ultimately influence objective health and socioeconomic mobility.

Compelling evidence from historical and contemporary academic research, as well as the spate of tragic events such as the murder of George Floyd and others, indicates that structural racism is a (if not the) major root cause of racial disparities in socioeconomic mobility and health (7, 29, 45, 103, 110). Structural racism is an organized system that differentially allocates opportunities to benefit certain racial groups (largely White and more affluent persons) while disadvantaging others (7, 100). We organize this review around a conceptual model that illustrates how pervasive structural racism influences racial disparities in health and socioeconomic mobility via pathways that include social capital, such as through social cohesion (Figure 1). Our conceptual model is embedded within the social milieu that concentrates and structures opportunity for some and concentrates inopportunity for communities of color, which are often lower resourced (53, 55, 70, 77).

The Black experience in America chronicles the nation’s history of oppression and the Black community’s efforts to resist, repair, and heal, which describes the Black American Freedom Struggle. One of the most poignant examples of how structural racism has a direct effect in thwarting social mobility among Black people in ways that have led to intergenerational suffering and poverty is the decimation of Black Wall Street, where 300 people died after 35 city blocks were burned by a White mob in Tulsa, Oklahoma. The Greenwood neighborhood was once the beacon for Black social mobility in the United States. What is the relevance of Black Wall Street in this discussion of social capital, social mobility, and public health? It is one story in a long history of the many ways that systemic oppression intended to destroy attempts by the Black community to organize and to develop strategies to resist injustice. On the 100-year anniversary of the destruction of Black Wall Street, the main culprits of the massacre and destruction of Black businesses have not been brought to justice and Black families have not received any compensation for the damages. Records of this event have been destroyed intentionally, many Black bodies were buried in mass graves, and the story of Black Wall Street has not been taught in classrooms locally or nationally. The fact that a major tragedy like this remains unresolved is eloquently summed up by W.E.B. Dubois:

The most difficult social problem in the matter of Negro health is the peculiar attitude of the nation toward the well-being of the race. There have … been few other cases in the history of civilized peoples where human suffering has been viewed with such peculiar indifference.

(24, p. 163)

A century after Tulsa, structural racism continues to influence socioeconomic mobility and health opportunities for Black people across every domain in society, including the education system, the health care system, the labor market, and housing markets, as well as political processes and institutions including voting laws. Structural racism produces inequalities by directing which opportunities and resources are available and how they are distributed. Structural racism therefore determines individual and community-level access to all forms of capital (e.g., human, financial, cultural, and social) (Figure 1). By contrast, Tulsa is a historical example of how Black communities have organized themselves to build and sustain economic and social capital (54).

Truncating opportunity in one domain (e.g., education) affects mobility in multiple other domains (labor markets, housing, consumer credit). For example, the persistent racial segregation of neighborhoods, combined with the financing of public schools through local property taxes, means that Black adolescents attend a very different type of school compared with White adolescents (31, 62, 65). Inner-city schools, especially those in predominantly Black neighborhoods (compared with those in predominantly White neighborhoods), are more likely to be under-resourced (e.g., fewer up-to-date textbooks, no extracurricular activities, no Internet access) because Black families tend to live in neighborhoods with a lower tax base and less political, social, and cultural capital with which to secure the resources they need (22, 55, 62). The deliberate speed or pace that was taken to desegregate schools after the *Brown v. Board of Education* decision helped to overturn remnants of *Plessy v. Ferguson* and opened pathways for many schools to become re-segregated decades later (64, 66).

## STRUCTURAL RACISM AND HEALTH DISPARITIES

Racial disparities in health are equally as stark as racial disparities in socioeconomic opportunity. During the first year of the coronavirus disease 2019 (COVID-19) pandemic, overall life expectancy in the United States dropped a full year from 78.8 years (estimated in 2019) to 77.8 years during the first half of 2020 (75). To put this number in perspective, American life expectancy fell by 0.2 years between 2014 and 2015, when the alarm was first raised about the steep decline in American health status caused by the deaths of despair (viz., social isolation, suicide, drug overdose, and alcoholism). However, even the dismal 1.0 year drop in life expectancy for the country as a whole was overshadowed by the precipitous drop for Black people (2.7 years).

The United States has continued to live up to the calls of the Ottawa Charter for Health Promotion in its response to the World Health Organization (WHO)’s Healthy Cities initiative, which illustrated and helped to geocode inequality in several areas by documenting that the lack of proximity to public transportation, public services, and high-quality jobs can lead to higher morbidity and lower life expectancy. When we review benefits to health care access such as to HIV treatment, the evidence clearly shows that access leads to adequate and timely HIV treatment, faster recovery from HIV, and better quality of life. Despite the unprecedented access to antiretroviral therapy in the United States, racial disparities in care have been attributed in part to the longer distances that Black people have to travel to receive therapy because they do not have access in their neighborhoods (76, 107). Longer commutes translate into days off from work and lost income to entire families owing to the opportunity cost, in addition to other further reductions in disposable income for expenses such as gas and car maintenance. Time itself is a privilege, and racism results in lost time that has harmed Black communities (35).

In turn, disparities in the distribution, access, and quality of socioeconomic resources such as education, income, and employment have been invoked as primary mechanisms to explain racial disparities in health outcomes. During the COVID-19 pandemic, Black Americans have been more likely to be represented in frontline, consumer-facing industries involving high person-to-person contact, which exposed them to SARS-CoV-2 (severe acute respiratory syndrome coronavirus 2); they are less likely to be working in occupations with the ability to switch quickly to telework; and they are more likely to be working in jobs that do not offer sick days or time off to receive the COVID-19 vaccine (88). Black Americans are also more likely than White Americans to live in segregated neighborhoods, with fewer spaces to socially distance, and in multigenerational households; more likely to be doubling up with other people (family, friends) as a result of job loss and eviction; and more likely to be exposed to air pollution, which increases the risk for serious infection. The depth of job loss in Black communities will resonate for many years. Similar patterns followed as Black business owners were left out of early rounds of Paycheck Protection Program loans (26). This deficit was felt by many Black businesses, which are concentrated in barber and beauty salons, lawn care, and social/community services.

## SOCIAL CAPITAL AS A DETERMINANT OF SOCIAL MOBILITY

Social mobility and opportunities for health are enhanced by access to a variety of forms of capital, e.g., education (“human capital”), income and wealth (“financial capital”), and the resources embedded within social connections (“social capital”). Social capital describes the collective actual or potential resources available through social connections or durable networks that individuals or groups can access as well as features of organizations that make it possible to achieve some coordinated or purposive action (8, 15, 68). Much of the popular understanding of social capital today is about its structural forms, including network-based elements, such as number and strength of social ties, or behaviors, such as participation in neighborhood activities (60, 69). A parallel body of research has emphasized elements of social cohesion, often captured by cognitive and attitudinal aspects such as perceptions of trust, feelings of belongingness, and perceived norms of reciprocity (59, 73). The common thread running through social capital and cohesion is the premise that there is value in the networks and that the norms that exist within networks produce expectations of actions, such as cooperation or reciprocity exchanges, which advance individual and collective goals.

Social capital can be conceptualized at multiple levels. At the macro level, social capital operates through relationships among states or jurisdictions (e.g., the tristate collaboration among New York, New Jersey, and Connecticut) or may even occur across entire countries (e.g., the commonwealth of Caribbean nations). The meso level, which has been the predominant focus of public health scholarship, operationalizes social capital within units such as residential neighborhoods, schools, and workplaces (46). Although it is less commonly analyzed in public health scholarship, microlevel social capital typically refers to the quality and quantity, density, range, and diversity of relationships, including close or loose ties to friends, family, and acquaintances (5).

## SOCIAL CAPITAL AND BLACK SOCIAL MOBILITY

Social capital has yet to occupy a prominent role in the debate about the drivers of racial disparities in socioeconomic mobility and health. Few studies have examined the role of social capital as a mechanism to redistribute power from the macro level (e.g., labor markets, housing, institutions of justice) down to the level of communities (13, 22, 108). The contributions of social capital—at least in the formal definitions we use above—have not always been explicitly articulated within major theories invoked to explain racial disparities in social mobility. Social capital needs to be taken seriously as a determinant of social mobility disparities between Black and White people because it, too, is influenced by structural racism.

To date, only a handful of studies have explicitly explored the effects of social capital and health disparities or inequality (14, 36) on the health of segregated populations. One study, by Hart (48), found that Blacks with greater contact with other Blacks at school, in the current neighborhood, and at church had higher mortality. But this study did not control for sociodemographic differences among study participants and used only self-reports of segregation (48). Another study, by Hutchinson et al. (52), found that Blacks living in predominantly Black neighborhoods with low social capital experienced lower mortality compared with Blacks living in predominantly White neighborhoods with low social capital. A more recent study of social capital in Jim Crow states found that even though Jim Crow laws were abolished in 1965, lower social connectivity, community, and trust were found in those states and that the association was compounded by lower income. Collectively, these findings suggest that the combination of physical isolation and social isolation due to segregation has deleterious effects on health that can be only partially mitigated by increasing social capital alone. On the one hand, by helping to secure access to resources for social mobility, increasing the stock of social capital is likely to be of critical importance for achieving health equity for Black Americans. On the other hand, the nature of these pathways is complex, and greater stocks of social capital may not always translate to greater social mobility or better health outcomes. The relationship between social capital and health often depends on the quality and type of linkages among people.

We can identify at least three dominant narratives to account for racial disparities in socioeconomic mobility and its relation to social capital. Although controversial when it was introduced in 1965, the first account, commonly known as the Moynihan Report, came from the US Department of Labor, Office of Policy Planning and Research (106). Fragmented (or broken) families were put forth as a primary causal agent for the persistent inequalities. As Moynihan described in the introduction of his report,

The fundamental problem, in which this is most clearly the case, is that of family structure. The evidence—not final, but powerfully persuasive—is that the Negro family in the urban ghettos is crumbling. A middle-class group has managed to save itself, but for vast numbers of the unskilled, poorly educated city working class the fabric of conventional social relationships has all but disintegrated.

(106)

The primary data that furnished the statistics for Moynihan’s account were rising rates of marital dissolution, the proportion of single female-headed households, illegitimate births, and welfare dependency. At the individual and family levels, social capital can be reduced through marital dissolution if resources for social and economic support are also broken or severely reduced. At the community level, social cohesion may also be reduced, owing to the collective psychological toll on families that may further reduce people’s opportunities to fully participate in neighborhood activities (82). While Moynihan implicated the historical legacy of slavery as the main antecedent cause of these problems, the primary mechanisms through which he linked male joblessness and poverty were in material terms—a wage system that was insufficient to sustain a family. Urbanization was also discussed as a problem. Many Black Americans during the Great Migration between 1910 and 1920 who fled the South to northern cities in search of work were forced into specific neighborhoods that had poor working, living, social, and environmental conditions and, because of racial residential segregation, had limited prospects of upward mobility.

Federal policies during the New Deal further entrenched the segregation and isolation of Black communities through the establishment of the Home Owners Loan Corporation, which redlined minority communities (70, 77, 113). In turn, the concentration of poverty across decades and generations produced a psychological effect structured by hopelessness that inhibits community engagement, trust in neighbors, and attachment to one’s neighborhood (34, 51). Another contributor to the erosion of social capital is spatial stigma—i.e., the social construction of attributing to place a lower value, negative characteristics, and other degrading symbols on the basis of preconceived structural biases (37, 61).

The second major explanation put forth for racial disparities in socioeconomic mobility is the disappearance of work through deindustrialization, as argued by William Julius Wilson (30, 111). For Wilson, the primary causal factors are macrolevel economic policies that drive changes in the organization of the city. Urbanization, deindustrialization, and White flight are linked to mobility outcomes through the deterioration of neighborhood social capital. Specifically, Wilson argued that in areas where Black people are united by a common struggle against inequality, the same strong social ties may limit mobility if tightly connected networks are not linked to economic or social opportunities (96). Royster’s (93) work examining blue collar workers offers up a clearer explanation: that racial discrimination is operating as a dominant, yet invisible force in determining how network connections operate differently for White male blue collar workers compared with Black men, opening up employment opportunities for the former while shutting out the latter.

Sampson’s work points to another way in which social capital formation might work to reduce socioeconomic mobility for Black Americans. Social connections, he argues, sometimes work by connecting people to the “underbelly of social life” such as gangs or street networks, which, paradoxically, are employed by some youth to develop social capital to buffer the realities of living in communities with high crime and high unemployment rates (10, 97, 98). Elijah Anderson (3) further details how violence may also act to preserve the social capital of a network, which in many ways contains “decent” people who are often forced to employ street code strategies to survive the structural realities of living in socioeconomically disadvantaged neighborhoods.

The third major force linking social capital to racial disparities in socioeconomic mobility is the phenomenon of mass incarceration. In her seminal work, Michelle Alexander (1) shows how the criminal justice system is a legacy form of racism that Jim Crow introduced. Jim Crow laws allowed the formal and informal policing of Blacks that stemmed from Fugitive Slave Laws and laws that relegated Blacks to industrial and agricultural jobs for low pay. These laws also kept many Black sharecroppers from pursuing legal means to be paid fair prices for agricultural products and prevented them from recouping land that was taken as payment when White businesses and landowners assessed Black sharecroppers’ crops and livestock at lower values. These laws, policies, and informal practices systematically thwarted economic opportunities for Black Americans.

Today, Black men have the highest rates of incarceration in the United States, contributing to their invisibility across various contexts (39, 80, 112). Many employers in the formal sector deny jobs on the basis of a prior conviction, automatically disqualifying many Black Americans from generating earnings. Sentencing laws instituted during the War on Drugs produced longer sentencing periods for Black offenders, leaving them outside of the workforce for longer periods and producing systemic joblessness. Another socioeconomic mobility trap resulting from incarceration policy is that families—especially the women who have children with men who were incarcerated—are sometimes forced to accept public assistance to survive the loss in family income, and some policies prevent men from returning to these homes.

The totality of the incarceration effects on social capital can be summarized through four primary pathways (74, 92). First, high joblessness among the formerly incarcerated constrains already scarce resources and weakens a sense of empowerment to strive. Joblessness also reduces bridging connections to outsiders who might be in positions of power to provide networking opportunities to Black families. Incarceration is also linked to internalized and externalized stigma, which may affect one’s identity and result in driving people away from their native communities and reduce trust among one’s social network. Finally, incarceration has a devastating influence on Black communities through reduced health and collective efficacy of the adult population to enforce positive social norms and social control, particularly among young adults (89, 99). While incarceration depletes social capital among the Black populations by removing youth and adults, the social capital stock is further depleted when parents, as a result of disrupted networks of care and fear of violence, relocate their children to other neighborhoods as a strategy to preserve their lives (56, 91).

## IMPLICATIONS FOR SOCIAL CAPITAL RESEARCH AND PRACTICE

### Addressing Structural Racism Through a Revised Social Capital Agenda

Despite the significant body of work connecting social capital to health and socioeconomic outcomes, there is a paucity of published scholarship specific to racial disparities in both outcomes (2, 25, 81). There are dark sides to social capital, where some groups benefit from the systematic exclusion of other groups—often on the basis of race, class, and geography—and which result in the inequitable patterning of health and social mobility outcomes through limited connections with people in power, fostering group intolerance, excluding some groups from resources, or even causing division and strife within marginalized communities over scarce resources (23, 72, 109).

### Applying Critical Race Theory and Praxis to Social Capital

We have discussed that structural racism is a primary distal factor that catalyzes differences in social capital and downstream effects through multiple causal pathways, ultimately influencing racial disparities in both health and socioeconomic mobility. Reasons for the limited research on race, social capital, and health and economic disparities are numerous and complex. Moving forward, we want to apply public health critical race theory and praxis (PHCR) to our understanding of social capital as it has operated within Black communities in the United States. To achieve this aim, we draw on the critical race theory literature (6, 16, 20, 27, 28).

Applying principles and tenets of PHCR requires us to exercise race consciousness, i.e., a deliberative consideration of the role of race as well as an awareness of racialized differences in society, in particular as they relate to health behaviors, health outcomes, and opportunities to access health-promoting resources (28). Adopting a race consciousness accomplishes several steps: It (*a*) facilitates a deeper understanding of historic and contemporary patterns of race relations; (*b*) provides a clear focus on how knowledge is constructed and produced as it relates to social capital and health; (*c*) restructures current approaches on measuring social capital and health by applying this race consciousness to measurement approaches; and (*d*) guides future directions on how a race consciousness influences public health strategies moving forward and how the study of social capital (e.g., theory and methods) should integrate theories and methods that capture the intersections of race, class, gender, sexual orientation, place, and policy. This approach sets the stage for why this work challenges previous worldviews, values, assumptions, and frameworks about how social capital has been defined, researched, analyzed, and related to health disparities (27, 38, 115). We provide concrete actions for how to activate a race consciousness approach in social capital research moving forward. These strategies can become ways to increase access and opportunities to social, economic, and health-promoting resources endemic within the Black community, and outside of Black communities, which structural racism has so far limited from reaching these communities.

### From Theory to a Race-Conscious Social Capital Agenda

The first step forward in a race-conscious social capital agenda is the need for newer theories that specifically challenge the values and assumptions inherent in several definitions (84). Most of the social capital theories that dominate the public health discourse (e.g., those of Putnam, Coleman, and Bourdieu) identify social capital as a means to achieve some end. However, those ends focus on some political or economic outcome. Rarely has social capital been appreciated solely as a means without some end in mind. Moreover, social capital has not been applied to or framed as a means to achieve other things such as healing or restorative justice, recognition, or legitimacy that might be essential to change the relationships between structural inequalities and economic or health outcomes for Black Americans.

New theories should also question the values placed on certain indicators and constructs that are results of both material and psychological privileges (e.g., of being White, male, upper class, a researcher at a predominantly White academic institution, and living in a high-resourced neighborhood) (84). As such, the refinement of theory will be a bidirectional process of influence on and from the methods of inquiry and measures used in studies, the statistical approaches, lessons learned from practice, and advocacy-based implications of research findings. The following four action steps are not exhaustive but are designed to stimulate the future imagination for this research. We provide action steps rooted in experiences and examples of Black life, survival, thriving, and resilience.

#### Action Step 1: Integrate specific components of Black culturally resonant indicators of social capital into existing scales as subscales.

Given that much of the social capital and public health work operationalizes social capital as a group-level construct, we limit the discussion here to scales and other measures that have been used to operationalize macrolevel processes. For example, several state- and county-level social capital measures have been developed and used in empirical research (67, 94). Some indices have included subscales that highlight social capital challenges that may be particularly pronounced in Black communities (e.g., including a family unity subindex that without proper context may further misconstrue issues identified in the Moynihan Report about fragmented families). Other measures, however, are now beginning to include equity-related concepts (e.g., race/income equality and race similarity). Nevertheless, all these measures still fall short of meaningfully integrating Black-specific or salient forms of social capital into the scales (105). For instance, these scales include a component of the density of religious organizations or are reported as per 10,000 persons. Yet, there is limited discussion in the theory to explicitly acknowledge forms of religious social participation that vary by race. Hence, studies do not differentiate between predominantly Black Protestant or other historically Black religious organizations that may be part of Christian and non-Christian denominations and organizations (e.g., Nation of Islam, Muslim). The heterogeneity within Black religious institutions may constitute a range of mainstream and nonmainstream institutions, such as evangelical Protestant or Catholic organizations, but they are not fully accounted for within the literature and in their relationship to social capital and/or health outcomes. The literature would be more robust with the inclusion of these institutions to highlight the documented differential levels of religious participation between Black and White people as well as the potential differences in their contextual influences on Black communities (as compared with other racial/ethnic groups) (47, 86, 87).

Creating forms of social capital that are salient for Black people requires including them in the process from the design to the measurement to the interpretation of the findings and in the decisions made regarding appropriate interventions (see the sidebar titled Case Study 1: The Flint Water Crisis). For instance, Dean and colleagues (19) recognized from their personal lived experiences and from discussion with community residents that block parties were a form of social capital. Conceptualized in this way, the authors found that people living in higher-density neighborhoods in Philadelphia, Pennsylvania, tended to have higher rates of cancer screening outcomes. In Figure 1, we provide several areas where one might identify existing metrics or develop new indicators of Black-specific or salient forms of social capital. For instance, civic and social participation measures should specifically ask about people’s participation in Black Greek letter organizations (i.e., fraternities and sororities) (79). Three of these organizations originated in the early 1900s on White campuses in response to the social isolation and exclusion that Black people faced on college campuses. Today, these organizations provide social support and community service, as well as a strong sense of racial identity, pride, and advocacy for Black communities.

In addition to Black Greek letter organizations, a range of other civic, social, and activist organizations emerged after the turn of the twentieth century (30, 41) during Reconstruction and in the late 1800s. Many of these organizations continued the pro-Black abolitionist movement and included the Black Women’s Club Movement, which launched many antilynching campaigns (50). Black physicians, nurses, and other health care professionals developed professional organizations because of racist views that kept them out of established White professional organizations (11, 12).

Social movements, including civil rights marches and protests, have long been vehicles for Black people to gather, mobilize, resist, and express concerns for change. Social movements and protests—initiated by Black people or joined in solidarity—are an underappreciated metric that captures bonding, bridging (even crossing international borders), and linking forms of social capital simultaneously [e.g., Black Power Movement, March on Washington, Black Lives Matter (BLM) protests], established to improve safety, police reform, social mobility, and health (38, 57). Therefore, researchers should consider using social movement and protest data either independently or in scales to acknowledge and value the contributions that each brings to the ongoing fight for equity and justice for all citizens in the United States and link them to health equity outcomes.

We should not stop here. Social capital, as we mentioned, is not always a means to some economic end, but rather for Black people, in particular, it has been a form of healing. One way to acknowledge the injury and assault on Black lives is to validate representation and protest as a form of healing rather than as dissent against law enforcement. Acknowledging protests as a form of social capital salient for Black people could start through the curation of reliable protest data, including indicators such as locations, density of the crowds, and length of days of previous protests (e.g., some of these data are currently available via Social Explorer). Quantifying those data will allow us not only to empirically link protests to changes in health and economic outcomes but also to anticipate the support and social, emotional, and mental health resources that protesters need.

While racial equity indices are emerging in social capital indicators, we need a more explicit focus on indicators of success and social enterprise around Black businesses (63). The socioeconomic opportunities of Black businesses are constrained by systemic racism through inadequate opportunities for business capital funding, including grants and loans. Moreover, Black people have less access to social capital networks of entrepreneurs and the mentoring that they need to start and maintain their own businesses (96, 111). We therefore need access measures that quantify the benefits, challenges, and inequities in navigating social, organizational, and regulatory networks, which Black businesses have historically experienced (and continue to experience). One potential metric to include is the density of Black-owned businesses, which is now a feature on Google Maps that labels businesses as “identifies as Black-owned.” These metrics should be verified through official sources from the Bureau of Labor Statistics, as well as from community residents and elsewhere, to track and identify growth, sustainability, or progress or lack thereof (e.g., increasing or decreasing trends) at multiple aggregate levels (e.g., in neighborhoods, community districts, counties, and states). Collecting this information is important to assess the short- and long-term impact of Black-owned businesses on social mobility and health. The development of monitoring and tracking systems can be used in traditional networking approaches and in newer technologies that can enhance social capital among Black business owners (e.g., quantifying the extent and quality of network links among owners in the same area). Local and federal government resources can be leveraged to develop the ecosystem of solidarity, community cohesion, and reciprocity needed for social mobility.

Next, farming has been a potent form of social capital in Black communities that will continue to be missed if traditional forms or conceptualizations of social capital remain. Monica M. White’s research highlights how Black Americans have reclaimed farming as a form of not only resistance but also economic sustenance and legitimate business pursuit for younger generations today. As this literature emerges, however, it will remain important to separate constructs of community gardening from Black sustenance farming because the former is not always associated positively with social processes that promote equity in Black communities (9, 43).

In addition, we need metrics that evaluate social capital opportunities at the school level, particularly for Black children. There is no shortage of scholarship documenting inadequate resources such as textbooks and extracurricular activities as a result of disinvestment and a lack of finances and how these inadequate resources impact health and mobility outcomes among Black children. However, greater research is needed to identify and develop forms of social capital that are most helpful for promoting intergenerational mobility in the Black family. Marion Orr’s work showed that some of the limits of “Black social capital” operationalized as a bonding intergroup coalition among Black parents and community members is not always sufficient to achieve desired outcomes because it may hamper necessary bridging and linking of social capital (78). These consequences point to the potential downsides or costs of social capital for some groups (81). However, an additional monetary cost of producing social capital will need to be acknowledged within the financial constraints faced by inner-city schools, the students, and the families from which they come. Crowley & Green (17) list a set of social capital activities and the potential monetary cost attached for activity per person, ranging from $0–$205 [e.g., establish a tool-lending resource ($17–$34) or host a block party ($34–$205)], which can be quantified across many levels and could be ranked and prioritized in order of importance.

#### Action Step 2: Question and even eliminate specific measures from validated constructs.

Specifically, Putnam (82) surmised that differences in social capital by race can occur only in areas where Whites exit integrated communities. This is one value judgment and assumption among several critiques that have been mounted on Putnam’s and other popular indicators of social capital in public health research (102). As Stolle & Hooghe state, “[W]e should not assume that patterns of sociability, generalized trust, and reciprocity develop as a matter of course” (101, p. 232). One construct to consider removing when investigating health disparities is voting (101). For example, voting, which is considered a structural indicator of linking social capital, has been long been acknowledged as an indicator of social capital. High voter registration rates are linked to higher social capital in White communities, but this association does not hold in Black low-resource communities (49). Voter turnout, on the other hand, has more recently shown a significant positive association with social capital for Black Americans (49), but the association is still considerably weaker than that for Whites. The discrepancy in this finding may be explained by how social capital has formed in response to structures of opportunity: Black Americans have been historically disenfranchised from registering to vote, and the passage of recent voter suppression laws in several US states is evidence that these tactics are still in use. Thus, voter registration itself may be more reflective of voter suppression than actual social capital; however, among those who are able to register, they may need to deploy social capital to overcome additional barriers to achieve turnout (e.g., churches organizing and providing rides to voting sites). Beyond structural indicators of bonding, bridging, and linking social capital, researchers should also ask whether cognitive or attitudinal forms of social capital are theoretically applicable in each analysis. For instance, Friedman and colleagues (32) conducted a case study in the Bushwick neighborhood of Brooklyn, New York, and showed that for residents in those communities, “trust among neighbors” was not the central factor that mattered for health. In fact, there was widespread distrust among community members and across institutions of power (e.g., the police). The authors found that social network ties and informal negotiations for behavior and social norms were more important for this community because they allowed residents to contest the realities of living in areas characterized by crime and a lack of opportunity but in ways that were empowering and acknowledged their agency in response to social conditions (32). The implication is that low trust may not always signal a lack of social capital especially in Black communities, so researchers should be cognizant of this fact and develop alternate competing hypotheses that support other causal processes in these specific contexts.

Granovetter’s (42) work on the importance of weak ties as a valid conceptual feature of social capital shattered the prior preconceived importance that was once ascribed to dense network ties as an indicator of strong social capital (97). Future research on social mobility and health disparities will therefore need to center, say, Black youth who are at the margins and contend with the importance of weak ties as it relates to social capital formation and consequences for Black people. For instance, drawing on longitudinal ethnographic studies of 15 Black males (ages 12–16) in central Harlem, Richardson & St. Vil (90) described a strategy called “rolling dolo,” which the young men used to distance themselves from some Black males in their community in order to protect themselves from crime and violence.

Beyond the development of new theory, scales, and indices, other corrective measures may be fruitful to pursue. An initial step is for researchers to avoid viewing Black spaces (e.g., churches, barbershops, beauty salons, community centers) and contexts as sites solely for recruiting Black participants and disengaging Black communities from participatory approaches. We are calling for participatory approaches in research that center Black communities, that restructure our research paradigms, and that provide adequate methodological space with which to integrate both quantitative and qualitative methods and situate them within community engagement activities. A full consideration of these methods can lead to more meaningful ways to assess, evaluate, and develop more effective and efficacious interventions that move toward health equity and do not identify causal factors of health disparities (40, 104).

As we have documented earlier, a focus on quantitative analysis alone will typically miss how social capital strategies are employed in complex ways that might seem contradictory, counterproductive, or contrary (in the dominant White-centered social capital lens) to health and mobility (e.g., uprooting children from one neighborhood and breaking their current social ties to save them or a mother viewing jail as a place for social resources for her son) (55, 90, 91). Participatory forms of research will enable us to bring to the forefront the ways in which social capital is produced in spaces of contested politics and rhetoric framed around disorganization (e.g., a focus on vandalism during BLM protests despite the majority of participants being peaceful) to understand how protest may be an end in itself—one that produces healing from multiple forms of state-sanctioned trauma that can be healed only through solidarity and vocal expression.

#### Action Step 3: Apply critical race theory and a race-conscious social capital agenda and call for psychometric approaches that consider reweighting scales on the basis of items of importance or testing differential item functioning of common scales.

For instance, research has shown that some items in the *Diagnostic and Statistical Manual of Mental Disorders* (DSM)-IV alcohol use disorder scales are more sensitive for Blacks than for non-Hispanic Whites, which has clinical implications for the scale’s association with health outcomes as well as for treatment courses (58). We are unaware of any published studies that have conducted such analyses across race for common social cohesion scales, which is one strategy that several authors of this team are currently exploring. Appropriate weighting strategies will also need to be identified depending on the level of analysis and the data. For instance, while individual race is typically adjusted for within a matrix of other confounders to produce aggregate neighborhood-level social capital, further weighting for factors such as the level of residential segregation could be applied to those aggregate measures (46). A second statistical strategy is to conduct effect modification or interaction analysis by race/ethnicity when available. This research may take the form of stratifying analysis when the exposure is generalized (e.g., social trust among all Americans) but the outcome is race-specific (e.g., rates of HIV among Black people) or of analyzing race-specific estimates of social capital in response to race-specific health or social outcomes to identify spillover effects of social capital from one group to another (85). A third statistical strategy includes adjusting potential competing hypotheses or mechanisms that specifically might erode social capital for Blacks. For instance, although we argue for including historically Black churches in social capital scales, when doing so is not feasible or if a different research question exists to examine or isolate a particular social capital item that does not include civic participation, one should typically adjust for social capital that might arise from nonsecular spaces such as churches (85). A fourth statistical strategy to advance this work is to examine the impact of social capital simultaneously on health and on social mobility outcomes through multivariate analysis or other techniques such as structural equation modeling, which can handle multiple dependent variables. As we have documented and as described in our conceptual model (Figure 1), the relationship between health and social mobility specifically in relation to social capital among Black people is nonlinear and codependent.

#### Action Step 4: Identify more case studies and center Black forms of social capital in action and in ways that highlight Black excellence and economic mobility and health.

Social capital also operates through cognitive appraisals that condition what is seen and valued. Although few historical artifacts remain that we can use to characterize Black social capital during the Black Wall Street era in Tulsa, Oklahoma, there remain ample contemporary examples if we are willing to look. In the recent scholarship by Putnam & Feldstein (82), the authors traveled across the United States in search of examples within a community that demonstrate social capital at work, including “making connections, establishing bonds of trust and understanding, and building community” (p.1). While their work includes some examples of social capital in communities of color (e.g., see chapter 4, The Dudley Street Neighborhood Initiative), there remains a glaring void of other case studies that could more poignantly illustrate Black social capital in action (see the sidebar titled Case Study 2: The Black Panther Party). Scholars of color and others in academia should contribute to developing case studies that highlight Black social capital in action.

## CONCLUDING DIRECTIONS AND ACTION STEPS

Tracing the history of how Black Americans have used social capital to achieve social mobility allows us to highlight specific pathways through which social capital has been used to address health inequities. We identified the ways that structural racism has limited and continues to limit access and opportunity for Black communities, writ large. We also clarified how social capital may differ in Black communities, as they developed unique and enduring strategies to resist slavery, Jim Crow laws, legal segregation and re-segregation policies, voter disenfranchisement, and the lack of institutions and systems to provide adequate education, health care, and jobs. Black communities have become the subsistence of or the foundation for the American Dream deferred, reimagined, and remixed (in hip-hop vernacular) to identify and practice what Dr. King framed as the Beloved Community. This article is not just a literature review for public health and social capital scholars, but an invitation for all to reimagine our social, economic, and political structures that point toward building community with racial and health equity as the vision, process, and end goal. We intend for this work to spur actions that lead to an American society where health equity is the social norm. To achieve this goal, the structures and forms of social capital in all communities must be anchored to economic, political, and social policies, systems, and structures. We call on actors and agents across and within systems and structures to engage in anti-racism practices, policy making, and system building.

## CASE STUDY 1: THE FLINT WATER CRISIS

In 2013, individuals in the predominantly Black city of Flint, Michigan, began to report on the water’s poor taste, smell, and discoloration, as well as skin conditions that arose about 18 months after city leadership took a cost-saving measure by switching the source of residents’ water supply. Lead levels in water flowing from the Flint River were more than 25 times the acceptable levels set by the US Environmental Protection Agency (EPA), which would have lasting mental and physical health effects on residents (44, 95). Michigan’s Civil Rights Commission (71, p. 2) has deemed the inaction of city leadership a “result of systemic racism” based on the city’s large population of Black and low-income residents.

In a demonstration of collective efficacy,

- Community residents united with bonding social capital to bring national attention to the issue, first, by showcasing jugs of contaminated Flint water to city officials in protest of the city leaders’ inaction and later by petitioning the EPA to launch an emergency federal response (which the EPA did not do); and
- Residents began an informational campaign to elevate the visibility of the issue to the national level, joining with local and national groups to build linking social capital (a form of social capital characterized by relationships between people who are interacting across institutionalized power or authority gradients in society) and to sue the city and state officials to ensure access to safe drinking water.

Several lessons were learned:

- Collective action can help disseminate information to shift the balance of power and resources to overcome a disparity;
- Different forms of social capital may be useful at different stages to advance social mobility;
- Social mobility achieved through linking social capital catalyzed access to power and resources in excess of what had previously been available to Black and low-income residents;
- Governmental accountability and reparation for racial injustice is necessary to overcome health disparities;
- Legal remedies (which can be mapped using legal epidemiology tools) can help guide us to examples of linking social capital; and
- In Flint, Michigan, residents won a settlement that required the city to replace the contaminated piping and sources, provide bottled water to residents, and offer health programs for residents who were adversely affected. Their work continues today to ensure that the conditions of the settlement are met.

## CASE STUDY 2: THE BLACK PANTHER PARTY

The Black Panther Party (originally named the Black Panther Party for Self-Defense) was a community-based organization founded in 1966 by Huey P. Newton and Bobby Seale in response to poor living conditions, lack of opportunity, and overpolicing in Oakland, California. The Black Panthers developed several survival programs designed to serve the needs of Oakland, California, by providing food, free health care services, and even an ambulance service in response to persistently segregated health care. The health activism by this group paved the way for attracting resources to low-income communities, and their free breakfast program became the prototype for the National School Lunch Program and the School Breakfast Program, which still operate today (21, 33).

Several lessons were learned:

- Responses to the denial of linking social capital have bolstered the bonding of social capital in Black communities;
- Social capital has led to innovations in health that Black communities have used to overcome health disparities; and
- Social capital has spillover effects that have benefited those beyond the Black community.
